# Populational landscape of INDELs affecting transcription factor-binding sites in humans

**DOI:** 10.1186/s12864-015-1744-5

**Published:** 2015-07-22

**Authors:** André M. Ribeiro-dos-Santos, Vandeclécio L. da Silva, Jorge E.S. de Souza, Sandro J. de Souza

**Affiliations:** PhD Program in Genetics and Molecular Biology, UFPA, Belém, PA Brazil; Instituto de Bioinformática e Biotecnologia, Natal, RN Brazil; Instituto Metrópole Digital, UFRN, Natal, RN Brazil; Brain Institute, UFRN, Av. Nascimento de Castro, 2155 - 59056-450, Natal, RN Brazil

**Keywords:** Transcription factor, Transcription factor-binding site, INDEL, Population genetics

## Abstract

**Background:**

Differences in gene expression have a significant role in the diversity of phenotypes in humans. Here we integrated human public data from ENCODE, 1000 Genomes and Geuvadis to explore the populational landscape of INDELs affecting transcription factor-binding sites (TFBS). A significant fraction of TFBS close to the transcription start site of known genes is affected by INDELs with a consequent effect at the expression of the associated gene.

**Results:**

Hundreds of TFBS-affecting INDELs (TFBS-ID) show a differential frequency between human populations, suggesting a role of natural selection in the spread of such variant INDELs. A comparison with a dataset of known human genomic regions under natural selection allowed us to identify several cases of TFBS-ID likely involved in populational adaptations. Ontology analyses on the differential TFBS-ID further indicated several biological processes under natural selection in different populations.

**Conclusion:**

Together, our results strongly suggest that INDELs have an important role in modulating gene expression patterns in humans. The dataset we make available, together with other data reporting variability at both regulatory and coding regions of genes, represent a powerful tool for studies aiming to better understand the evolution of gene regulatory networks in humans.

**Electronic supplementary material:**

The online version of this article (doi:10.1186/s12864-015-1744-5) contains supplementary material, which is available to authorized users.

## Background

Much has been debated about the evolutionary role of genetic alterations in the regulation of gene expression [1–7]. In that aspect, transcription factor binding sites (TFBS) have recently been studied both in humans and other animals [8–10]. Several genome-wide analyses have identified regions close to genes (usually enriched with TFBS) showing patterns of diversity in accordance with a model of positive selection [1, 10]. In a recent study, Arbiza *et al.* [1] found that TFBS are under weaker selection than protein-coding regions of genes although these authors could observe several instances of adaptation in TFBS. In a similar way, Vernot *et al.* [10] have found hundreds of variations that are adaptive.

Although these studies have shed some light on the evolutionary forces acting on TFBS and other regulatory elements, several issues remain poorly explored or even unexplored. One of them is the role of INDELs (insertion/deletion) as a source of genetic variability among TFBS. Most of the few populational studies in this area are biased towards single nucleotide variants (SNV) [3, 9, 11]. Based on that, we decided to explore this issue by using three types of data recently made public. First, whole-genome sequences of more than a thousand human individuals from the 1000 Genomes Project (TGP) [12] were used to identify polymorphic INDELs. Second, a genome-wide identification of TFBS for 148 transcription factors from the ENCODE (Encyclopedia of DNA Elements) Project [13] was used to generate a catalogue of TFBS in the human genome. Finally, expression data from a sub-set of individuals from the 1000 Genome Project [14] was used to evaluate the impact of TFBS-affecting INDELs (TFBS-ID) on the expression of the corresponding gene. Integration of all these data allowed us to show a high frequency of TFBS-ID in the human genome. Hundreds of TFBS-ID showed a differential frequency in human populations and ontology analyses of these cases suggested a role of natural selection and population history in their distribution. Based on that, we argue that a TFBS-ID has been selected in Africans by down-regulating *APIP* (APAF1-interacting protein) and generating a better response to *Salmonella* infection. A comparative analysis with genomic regions, known to be under positive selection [15], revealed that a significant fraction of the TFBS-ID identified by us represent instances of adaptation in human populations.

## Results and Discussion

### Identification of TFBS-ID

**Fig. 1:**
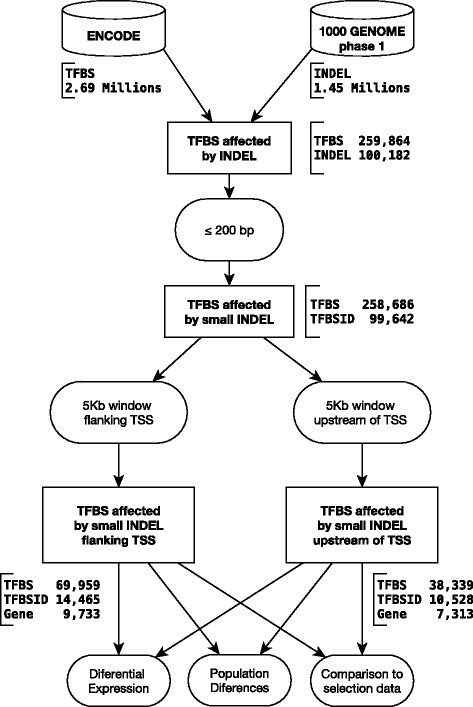
Analysis overview. Schematic representation of the strategy used here to identify and analyse TFBS affected by polymorphic INDELs in human populations

Fig. 1 shows a schematic representation of the computational pipeline used in all analyses reported here. To build a catalogue of TFBS-ID, we first indexed all TFBS identified by the ENCODE project in the human reference genome (hg19 version). Data from the 1000 Genomes project regarding the position of INDELs in the reference genome was then compared to the position of TFBS and those cases in which an INDEL overlapped with a TFBS were selected. This strategy rendered us a total of 259,864 TFBS affected by at least one INDEL. Since a significant fraction of TFBS overlap at the sequence level, the non-redundant number of TFBS-ID in the above set was 100,182 (an average of 2.59 TFBS per INDEL). Due to the presence of long INDELs affecting many TFBS at once, we decided to limit our analysis to those INDELs shorter than 200 bp, which gave us a total of 99,642 TFBS-ID and 258,686 TFBS. Although the superior limit was set to 200 bp, the final set of 99,642 TFBS-ID is strongly biased towards shorter indels. More than 99.8 % of all indels were equal or shorter than 20 bp. Next, TFBS-ID close (≤5 KB) to the transcription start site (TSS) of known human genes (as defined by the Reference Sequence set) were selected. In total, 7,313 human genes had at least one TFBS affected by a polymorphic INDEL in the 1000 Genomes dataset. This set of 7,313 genes had a total of 38,339 TFBS affected by INDELs and 10,528 TFBS-ID. A complete list of this dataset is available at Additional file 1: Table S1. Since many reports have also used a window that flanks the TSS of known genes [16,17], we have also defined a different window of same size (5 KB) now encompassing 2,5 KB in each side of a given TSS. For this window, we found that 9,733 human genes had at least one TFBS affected by a polymorphic INDEL in the 1000 Genomes dataset (with a total of 69,959 TFBS affected by indels and 14,665 TFBS-ID). The complete dataset found for the 5 KB window flanking TSS can be found at Additional file 2: Table S2.

**Fig. 2:**
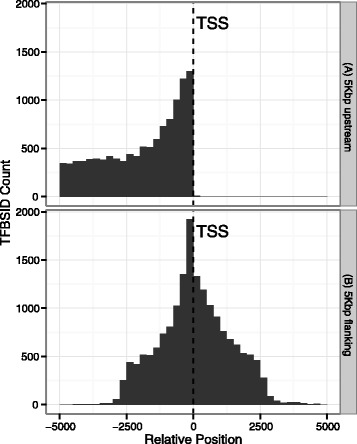
Relative position of TFBS-IDs within the 5 KB window adjacent to TSS of human genes. Overall distribution of TFBS-ID in both the 5 KB window upstream of TSS (A) and the 5 KB window flanking TSS (B)

TFBS-ID showed a biased distribution in terms of location within both 5Kbp windows proximal to the TSS of known genes. As seen in Fig. 2, their distribution tend to be closer to the TSS of genes (Fig. 2a) (the 3’ end of the 5Kbp window upstream of the TSS) while in the window with the TSS at center the distribution of TFBS-ID is symmetrical with a slight higher frequency at the upstream half of the window (Fig. 2b). When we split the TFBS-ID per type of transcription factor, the same biased distribution is observed for both windows, especially for some transcription factors (Additional file 3: Figure S1).

We were also interested in knowing what types of TF were more frequently interrupted by INDELs. A Monte Carlo simulation was performed testing the enrichment of specific TF within our TFBS final sets. Table 1 lists the top 20 transcription factors enriched for binding sites near genes (both 5 KB windows) and affected by INDELs compared to all TF binding sites near genes. Some of the TFs shown in Table 1 have already been identified in other analyses. Yokoyama *et al.* [3], for example, have recently shown that hominid-specific binding sites for *GATA1* and *CTCF* are enriched near genes related to sensory-related function and neurological pathways. *CTCF* binding sites have also been shown to be under positive selection in several Drosophila species [18]. *POL2* has also been studied in humans and chimpanzees by Kasowski *et al.* [8] who found inter-species divergence in the respective binding affinities.

**Table 1:** Transcription factors enriched in the set of TFBS-ID close to the TSS of known human genes. “TF” refers to the name of the transcription factor; “Number of TFBS” refers to the number of binding sites for the respective TF within the TFBS-ID set; “p-value” refers to the degree of significance for the respective TF enrichment with the final TFBS set against all TFBS near genes.

TF	5Kbp upstream		5Kbp flanking	
	N	p-value	N	p-value
*Pol2*	1818	<10^-4^	4505	<10^-4^
*CTCF*	1368	<10^-4^	1982	<10^-4^
*TBP*	879	<10^-4^	1941	<10^-4^
*HA-E2F1*	684	<10^-4^	1877	<10^-4^
*NFKB*	666	<10^-4^	1244	<10^-4^
*ZNF263*	606	<10^-4^	1238	<10^-4^
*TCF4*	370	<10^-4^	623	<10^-4^
*AP-2alpha*	237	<10^-4^	475	<10^-4^
*Pol2(b)*	364	0,002	802	<10^-4^
*YY1_(C-20)*	580	0,097	1134	<10^-4^
*Max*	511	0,162	962	<10^-4^
*CEBPB*	721	0,217	929	<10^-4^
*Pol2-4H8*	1264	0,246	2532	<10^-4^
*SP1*	534	0,315	1090	<10^-4^
*TAF1*	905	0,577	2120	<10^-4^
*USF-1*	538	0,668	872	<10^-4^
*CCNT2*	385	0,752	915	<10^-4^
*ELF1_(SC-631)*	693	0,993	1517	<10^-4^
*c-Myc*	613	0,998	1164	<10^-4^
*HEY1*	827	1,000	1523	<10^-4^
*Sin3Ak-20*	505	1,000	1129	<10^-4^
*E2F6_(H-50)*	524	1,000	1026	<10^-4^
*YY1*	434	1,000	872	<10^-4^
*GATA-1*	432	<10^-4^	747	1,000
*AP-2gamma*	351	<10^-4^	684	1,000
*GATA-2*	373	<10^-4^	546	1,000
*ELK4*	155	<10^-4^	450	1,000
*KAP1*	280	<10^-4^	444	1,000
*STAT1*	202	<10^-4^	359	1,000
*ZZZ3*	28	<10^-4^	41	1,000
*SETDB1*	193	<10^-4^	246	0,494
*TR4*	97	0,001	225	1,000
*E2F4*	216	0,001	506	1,000
*eGFP-GATA2*	95	0,004	127	1,000

### Evaluation of the effect of TFBS-IDs in the expression of corresponding genes

It has been shown that even small changes, like SNVs, in TFBS affect the affinity of the corresponding transcription factor and consequently the expression of the associated gene [8]. Therefore, we wondered whether the presence of an INDEL affecting at least one TFBS would change the expression pattern of the corresponding gene. RNA-Seq data for 465 individuals (all of them from the 1000 Genomes project) from the Geuvadis initiative [14] was used to compare expression and genotype data for the same individual. A statistical analysis was performed to identify those genes whose presence of a TFBS-ID was associated to a change in its expression (comparing individuals according to their genotype: homozygous for the absence of an INDEL, homozygous for the presence of an INDEL and finally heterozygous individuals). Out of the 7,313 genes with at least one TFBS-ID in the 5 KB window upstream of TSS, 6,248 were informative for this expression survey. Out of these 6,248 genes we found that 18.5 % (1,155 genes considering q-value ≤ 0.05 as a threshold) had its expression affected by the presence of a TFBS-ID (again by comparing individuals homozygous for absence of the TFBS-ID, homozygous for the presence of the TFBS-ID and finally, heterozygous). This is significantly higher than expected by chance (p-value < 10^-5^; OR 1.16). For the window flanking the TSS, we found that 1,804 genes (q-value < = 0.05) had its expression affected by the presence of a TFBS-ID (18.4 % of the total). Again, this is significantly higher than what one expect by chance (p = 0.04; OR 1.09). It is important to emphasize that the effect of the INDEL in the expression of the corresponding gene is certainly underestimated by our analysis since only one cell type was evaluated regarding expression. If a given gene is not expressed in the limphoblastoid cell lineage, no differential expression could be detected. The same is true regarding the expression of a given transcription factor whose binding site was affected by the INDEL.

What type of change is observed in the genes associated with a TFBS-ID? For the 5 KB window upstream of TSS, out of the 1,155 genes whose expression was changed, 654 were up-regulated and 553 were down-regulated in the individuals carrying a certain TFBS-ID, a significant difference from the null expectation (*p-value* < 0.01; OR 1.06). We could not observe any difference between the two datasets (up-regulated and down-regulated genes) regarding the type of transcription factors whose binding sites were affected by INDELs (*q-value* > 0.3). For the 5 KB window flanking the TSS, we found 990 and 912 up and down-regulated genes, respectively (a significant difference, p-value = 0.04). Like for the 5 KB window upstream of TSS, there was no enrichment of any specific transcription factor in either gene set (up or down-regulated – q-value =0.6). In both situations, the sum of up and down-regulated genes does not match the total number of differentially expressed genes because few genes are present in both lists, due to their different behaviour depending on the composition of subjects with a given genotype.

### TFBS-affecting INDELs with high differentiation between human populations

We next wondered whether we could identify in our set of TFBS-ID alleles that present a high differentiation between human populations represented in the 1000 Genomes Project. These frequency differences between populations are considered signatures of geographically restricted selection and have been used previously to identify regions under positive selection [13,19]. We restricted this analysis to a set of 911 individuals representing the three major continental groups: 246 Africans (AFR), 379 Europeans (EUR) and 286 Asians (ASN). To identify those INDELs with high differentiation between populations, we calculated the minimal frequency differences (δ) of the derived alleles between all pairs of populations and took into consideration all differences ≥ 20 % (δ ≥ 0.2). This threshold was based in statistical analysis of the distribution of all δ reported here, in which 20 % represents about two standard deviation from the mean (Additional file 4: Figure S2).

For the TFBS-ID identified in the 5 KB window upstream of TSS, this analysis generated a set of 1109, 507 and 663 TFBS-IDs that have a significant δ in AFR, EUR and ASN, respectively. When expression data is taken into consideration, 346, 149 and 132 TFBS-ID (out of the numbers above) seem to affect the expression of the corresponding genes in AFR, ASN and EUR, respectively. Table 2 reports the top 10 TFBS-ID with highest differentiation for all three populations. A complete list is presented at Additional file 5: Table S3. For the TFBS-ID identified in the 5 KB window flanking TSS, we found 1482, 679 and 885 that have a significant δ in AFR, EUR and ASN, respectively. A complete list for the TFBS-ID identified in the 5 KB window flanking TSS is presented at Additional file 6: Table S4.

**Table 2:** TFBS-ID within the 5 KB window upstream of TSS and with highest δ in AFR, ASN or EUR.

Population	dbSNP id	Gene	Type	Size	Population Frequency	δ
**AFR**	rs113103282	*CMAHP*	DEL	1	0.88	0.71
	rs111659599	*TMEM14C*	DEL	6	0.73	0.70
	rs201685762	*ATP1A1OS*	DEL	3	0.75	0.69
	rs200228600	*ATP1A1OS*	DEL	2	0.83	0.68
	rs60963584	*SAMD4B*	INS	1	0.79	0.68
	rs34107968	*MASP2*	DEL	3	0.08	-0.67
	rs3842412	*MIR6805, RPL28, TMEM238*	DEL	14	0.19	-0.66
	rs201075641	*ATP1A1OS*	DEL	4	0.71	0.65
	rs60602324	*IQCG*	INS	1	0.88	0.65
	rs59484263	*RESP18*	DEL	1	0.89	0.64
**EUR**	rs28366020	*NCDN*	DEL	3	0.06	-0.62
	rs5840961	*RP5-1004I9.1*	INS	1	0.08	-0.57
	-	*RP5-1004I9.1*	INS	1	0.08	-0.55
	rs34313783	*CELA3B*	DEL	1	0.62	0.53
	rs66822811	*DUT*	DEL	38	0.78	0.52
	rs5820777	*FAM117A*	DEL	1	0.66	0.50
	rs200692689	*MRPL36*	INS	2	0.88	0.49
	-	*MRPL36*	INS	1	0.88	0.49
	rs199953326	*MRPL36*	DEL	1	0.88	0.49
	rs75077631	*F12*	INS	1	0.77	0.49
**ASN**	rs201884277	*CCNL2*	DEL	2	0.87	0.75
	rs75244934	*MIR6808*	DEL	2	0.83	0.69
	rs139938620	*TAS1R3*	DEL	13	0.79	0.68
	rs34692283	*ADAT1*	DEL	2	0.74	0.67
	rs55726149	*EZR-AS1*	INS	3	0.20	-0.60
	rs77949675	*PHLDA1*	DEL	2	0.78	0.60
	rs35231579	*BAHCC1*	DEL	1	0.16	-0.58
	rs139775692	*ACAP3, PUSL1*	DEL	11	0.79	0.58
	rs61077744	*PYY*	DEL	1	0.70	0.57
	rs149347369	*FLJ42351*	INS	5	0.79	0.55

One interesting gene found in our analysis is *MC1R*, known to be associated with skin pigmentation in humans [20,21]. A TFBS-ID (rs201097793) associated to this gene has a higher allelic frequency in AFR (0.70) and ASN (0.64) when compared to EUR (0.17). This supports the suggestion from Vernot *et al.* [10] that regulatory polymorphisms, under recent selection, have an influence in pigmentation phenotypes. Another gene reported to have a TFBS-ID with a differential frequency is *VDAC3*, a voltage-dependent channel essential for sperm mobility [22]. We found a TFBS-ID (rs145074200) with a higher frequency in AFR (δ = 0.26), as similarly reported by Colonna *et al.* [23] for a different polymorphism in the same gene.

Taste perception has been crucial in human evolution especially for the detection of toxins. Not surprisingly, bitter taste receptors have been show to be under positive selection in human populations [24]. Our analysis (Table 2) shows that a TFBS-ID associated with *TAS1R3*, a sweet receptor, shows a high δ in ASN. Shi and Zhang [25] concluded, based in a comparison of several vertebrate species, that both bitter and sweet receptors are under positive selection. *TAS1R3* is also a component of the dimeric protein *TAS1R1/TAS1R3*, which is the umami taste receptor [26]. The umami taste is a common feature of many foods in Asia and is reasonable to speculate that this variant is being selected in Asians [27].

**Fig. 3:**
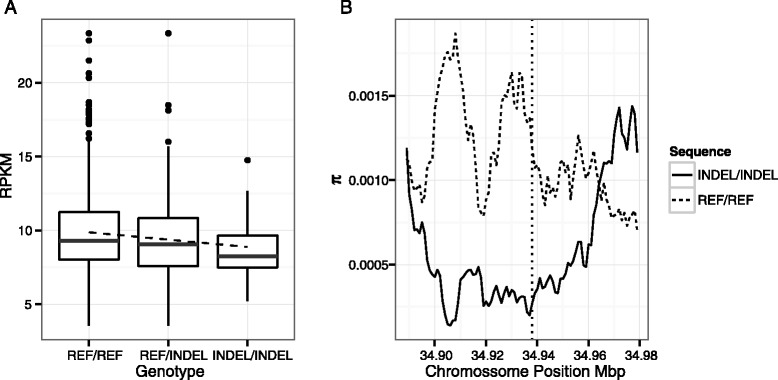
APIP expression is likely adapted in AFR. A TFBS-ID (rs139999735) with a δ = 0.22 in africans and associated with the *APIP* gene affects gene expression as seen in A. In B, individuals homozygous for the TFBS-ID (continuous line) had a lower genetic heterogeneity around the INDEL position (vertical dashed line) when compared to individuals homozygous for absence of the INDEL (dashed line)

Response to parasites and microbes has been constantly subject to adaptations in human evolution [28,29]. We found a TFBS-ID (rs139999735) with a higher allelic frequency in AFR (0.34 compared to 0.11 in ASN and 0.12 in EUR). The gene associated with this TFBS-ID is *APIP* (APAF1-interacting protein) whose protein has been shown to be an inhibitor of pyroptosis and apoptosis, both a response to Salmonella infection [30]. Based on that it is predicted that the TFBS-ID would cause a decrease in *APIP* expression. Indeed, Fig. 3A shows that expression of *APIP* decreases significantly in individuals homozygous for the TFBS-ID (*r*_*s*_ -0.13; p-value = 0.012). We propose here that this TFBS-ID is under positive selection in AFR due to a down-regulation of *APIP1* and consequently a better response to Salmonella infection. Fig. 3B shows that a selective sweep analysis supports this proposal. Individuals homozygous for the presence of the TFBS-ID show a decreased genetic heterogeneity around the TFBS-ID position (vertical dashed line in Fig. 3B).

**Fig. 4:**
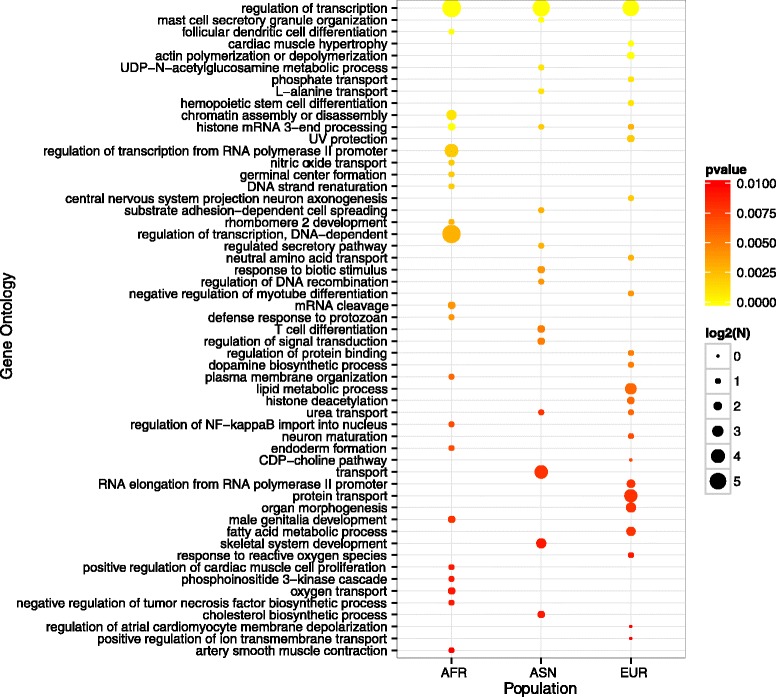
Ontology analysis for genes associated to TFBS-ID with δ > = 0.2 in the respective population (5 KB window upstream of TSS). Color of the circle refers to the p-value of the enrichment while size of the circle refers to the numbers of genes within that GO category

To gain further insights on what types of genes are associated with TFBS-ID showing a high differentiation between the three human populations, an ontology analysis was performed. Fig. 4 shows the major GO categories enriched (using a threshold of *p* ≤ 0.01) in the dataset of genes associated to TFBS-ID for each of the three populations used in this study (5 KB window upstream of TSS). Two GO categories were enriched in all three populations: “Regulation of Transcription” and “Histone 3’ end mRNA processing”. “Urea transport” is enriched in both ASN and EUR. All the other categories are enriched only in one population, as seen in Fig. 4. Overall, there are a large number of categories related to immunological response. Interesting categories enriched in Africans and Asians are “Response to protozoans” and “Response to biotic stimulus”, respectively. In Europeans one enriched category is “UV protection”, known to be under positive selection in this population [31]. For the 5 KB window flanking the TSS, some of the categories seen for the 5 KB window upstream of TSS are still present (Additional file 7: Figure S3) although several categories clearly linked to recent selection in humans are missing.

### TFBS-ID match regions known to be under positive selection in the human genome

In the last few years, several genome-wide strategies have been used to identify regions in the human genome that are under positive selection [15,28,29,32,33]. The recent availability of data from the 1000 Genomes project has catalysed such approach and hundreds of regions have been identified. To evaluate whether our set of TFBS-IDs correspond to genetic units that are under selection, a comparison was made with one of the most complete, in terms of the number of metrics used, of such studies [15].

**Fig. 5:**
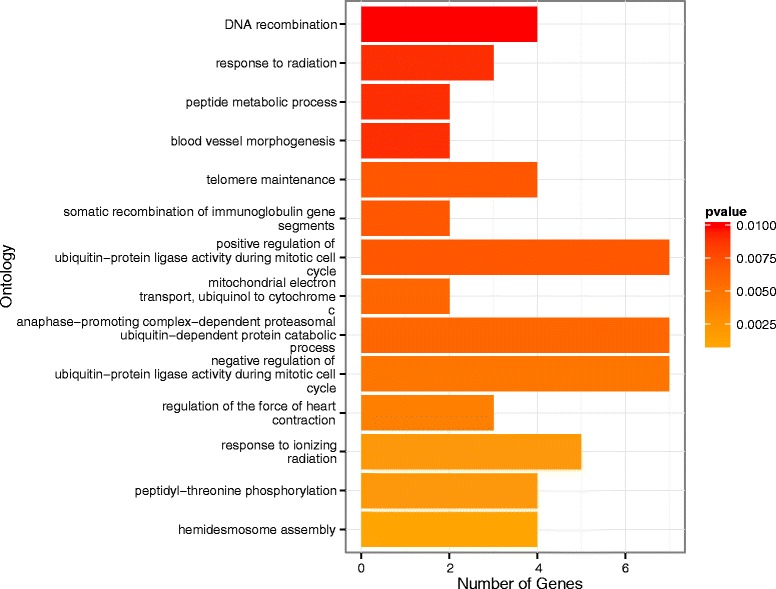
GO enrichment analysis for TFBS-ID matching regions known to be under selection in the human genome (5 KB window upstream of TSS). Color of the bars refers to the p-value of the respective enrichment. Length of the bar refers to the number of genes within the respective GO category

When we compared our total set of 10,520 TFBS-ID close to the 7,313 human genes (5 KB window upstream of TSS), we found that 3,499 (33.2 %) matched regions under selection as defined by Pybus *et al.* [15] within a 95 % confidence interval. With a 99 % confidence interval, we found 797 TFBS-IDs (7.5 %) that matched genomic regions under selection. For the 5 KB window flanking TSS, we found that 4,747 (32.3 %) TFBS-ID match regions under selection within a 95 % confidence interval. With a 99 % confidence interval we found 1,061 (7.2 %) TFBS-ID matching regions under selection. Fig. 5 shows the results for a gene ontology enrichment analysis (*p* ≤ 0.01) with the set of 797 TFBS-ID (5 KB window upstream of TSS) that matched genomic regions under selection. Three major categories are evident: ontologies associated with immunological responses, response to radiation and haematological/cardiac processes. All these processes have been shown to be under recent positive selection in humans [15, 23, 28, 31, 32, 34]. For the set of 1,061 TFBS-ID matching genomic regions under selection and within the 5 KB window flanking the TSS, we found while some categories are still present, when compared to the 5 KB window upstream of TSS, several differences exist (Additional file 8: Figure S4). Overall, the gene ontology analysis presented here (Figs. 4, 5, Additional file 7: Figure S3 and Additional file 8: Figure S4) suggests that the inclusion of a region downstream of TSS diluted the selection signal observed for the 5 KB window upstream of TSS. This in in accordance with a recent finding from the GTEx Consortium about a higher frequency of eQTLs located upstream of TSS [17].

## Conclusion

By integrating different types of data, we provide a comprehensive catalogue of polymorphic INDELs affecting TFBS in the human genome. Overall, our findings support the notion that regulatory variation has been important during human evolution. Some of the genes associated with these TFBS-affecting INDELs have been previously identified as targets of positive selection in human populations. The remaining set of genes and INDELs, however, represents a rich source of new information related to human evolution. We envisage that this dataset, together with the ones previously reported, will catalyse a series of new investigations on how recent human evolution has shaped gene regulatory networks.

## Methods

### Data Sources

Data from several projects were gathered in a local processing server for further analysis. This data included: (i) genome coordinates of all TFBS peaks from the ENCODE project [13] release 2 obtained from http://genome.ucsc.edu/encode; (ii) phase 1 genotype data from the 1000 Genomes Project Consortium [12] obtained from http://www.1000genomes.org; (iii) gene expression quantified by the Geuvadis project [14] obtained from http://www.geuvadis.org; (iv) the genome-wide selection measures of CLR [34], Fay and Wu's H [35], Fu and Li's D [36], R2 [37] and Tajima's D [38] calculated by Pybus *et al.* [15] obtained from http://hsb.upf.edu/; and (v) genome coordinates of the largest transcript of each known human gene from RefSeq release 64 obtained from http://genome.ucsc.edu/. All the data from humans was obtained from public sources. All ethical considerations were dealt in the original publications.

To identify TFBS peaks, ENCODE project analysed ChiP-seq of 145 TF's antibodies among 95 cell linages employing a pipeline developed by Landt *et al.* [39]. This pipeline uses multiple peak calling software (e.g. MACs, SPP and PeakSeq) and analyses replicates variance to further improve the peak calling sensitivity [13,39]. The employed procedure is detailed at the ENCODE project guideline page (http://genome.ucsc.edu/ENCODE/experiment_guidelines.html).

This study is exempted from ethical approval since all human data used here is publicly available in an anonymized fashion.

### Data Filtering and Annotation

Using GATK v.2.6 [40] (Genome Analysis Toolkit) we first filtered all INDEL variants shorter than 200 bp reported by TGP that overlapped an autosomal TFBS described by ENCODE. It is important to mention that our pipeline establishes as a rule that the beginning of a given indel had to be inside a TFBS, precluding therefore that a whole TFBS be removed by an indel. This set was then annotated and only those TFBS-ID near any known gene (up to 5Kbp upstream to TSS) were selected using snpEff v.3.5 [41]. This procedure and the number of elements at each step of the pipeline are illustrated in Fig. 1. The results were organized in a local MySQL v.5.5 (Oracle Corporation) database for easy access and manipulation.

### Statistical Analysis

All statistical analysis and plotting were performed with R package v.3.1 [42]. Multiple analyses were corrected by Benjamin-Hockberg method (or False Discovery Rate - FDR).

### Population Differentiation

To identify differentiated alleles among the European, Asian and African populations from the TGP (376, 286 and 246 individuals respectively), the minimum allele frequency difference (δ) for each mutation per population was calculated according to the following equation.

$$ \delta \left(i,j\right) = \min \left(\left|{f}_{i,j}-{f}_{i,k}\right|\right)\forall \mathrm{k}\in \left\{\mathrm{P} - \mathrm{j}\right\} $$

Where δ(*i,j*) is the minimum allele frequency difference of the variant *i* in the population *j*; *fi,j* is the allele frequency of the variant *i* in the population *j*; *f*_*i,k*_ is the allele frequency of the variation in the population *k* and P is a representation of all populations investigated (in this case EUR, ASN and AFR). This analysis did not include the American samples from the TGP due to their admixed nature.

### Gene expression association

The Spearman correlation test was used to evaluate any putative association between genotype data from TGP and gene expression data from Geuvadis of all TFBS-ID associated to the respective genes. The number of variant copies was assumed as dependent variable (therefore 0 for reference homozygous, 1 for the heterozygous and 2 for mutant homozygous). The same is true for the gene expression measured in FPKM (Fragments per Kilobase of Transcript per Million Mapped Reads). To interactively perform this test, a Python v.2.6 (Python Software Foundation) script was developed using SciPy v.0.14 [43] statistics library to calculate the correlation. The result was later filtered for non-quantified genes and non-variable genotypes among the Geuvadis samples.

The spearman correlation coefficient was calculated using the following formula (r_s_), where *n* is the sample size, r_variants_ is variant number rank and r_fpkm_ is fpkm rank. Rank ties were resolved using rank tie mean value. The correlation p-value was obtained by approximation to a t distribution and multiple testing was corrected by Benjamin-Hockberg method. The correlation was considered significant on q-value ≤ 0.05.

$$ {\mathrm{r}}_{\mathrm{s}} = 1\ \hbox{--}\ \left[6\ \sum\ {\left({\mathrm{r}}_{\mathrm{variants}}\hbox{--}\ {\mathrm{r}}_{\mathrm{fpkm}}\right)}^2\right]\ /\ \left({\mathrm{n}}^3\hbox{--}\ \mathrm{n}\right)\Big] $$

### Gene ontology category enrichment

To evaluate potential functional aspects within the set of investigated genes, we analysed gene ontology enrichment by two strategies. The first one employed ClusterProfiler v.2.0 [44] from the R package to search for overrepresented categories on the subset of investigated genes based on hypergeometric distribution. The second strategy employed a Monte Carlo method to evaluate the probability of ontology enrichment using 10,000 random simulations.

During each Monte Carlo simulation, a random gene set was generated with the same size of the investigated set and its ontology annotated. The ontology p-value was obtained from the simulated distribution of annotated genes.

### Positive selection sites identification

To identify genomic sites under positive selection, we gather data of five statistical measures of positive selection (CLR, Fay and Wu's H, Fu and Li's D, R2, and Tajima's D) [34–38] from three populations (Utah Residents with Northern and Western European ancestry; Han Chinese from Bejing, China; and Yoruba from Ibadan, Nigeria representing EUR, ASN and AFR populations respectively) calculated by Pybus *et al.* [15]. All five measures are common positive selection score of the literature further explained on [15,34–38]. The authors computed a ranked p-value according to each measure genome-wide distribution to access the value statistical significance. The ranked p-value was obtained by sorting the measures genome-wide scores and computing the fraction of higher scores, further explained on Pybus *et al.* [15]. Since each measure considers different selection parameters, any given site was considered under selection with at least 95 % confidence interval (*p-value* < 0.05) to any measure or population.

## Additional files

Additional file 1: Table S1. TFBS-ID description and associations for 5 KB window upstream of TSS. Additional file 2: Table S2. TFBS-ID description and associations for 5 KB window flanking TSS. Additional file 3: Figure S1. Distribution of TFBS-ID within both 5 KB windows split by types of transcription factor. Additional file 4: Figure S2. TFBS-ID minimum frequency difference (δ) distribution. The black curve represents the observed δ distribution and the red curve represents a normal curve of same mean and standard deviation. The dashed lines indicate two standard deviation (~0.2) from average (regarding the observed δ distribution) on both sides. Additional file 5: Table S3. TFBS-ID showing δ > 0.2 between populations (5 KB window upstream of TSS). Additional file 6: Table S4. TFBS-ID showing δ > 0.2 between populations (5 KB window flanking TSS). Additional file 7: Figure S3. Ontology analysis for genes associated to TFBS-ID with δ > = 0.2 in the respective population (5 KB window flanking TSS). Color of the circle refers to the p-value of the enrichment while size of the circle refers to the numbers of genes within that GO category. Additional file 8: Figure S4. GO enrichment analysis for TFBS-ID matching regions known to be under selection in the human genome (5 KB window flanking TSS). Color of the bars refers to the p-value of the respective enrichment. Length of the bar refers to the number of genes within the respective GO category.

## Abbreviations

AFR: African population from 1000 Genomes Project
ASN: Asian population from 1000 Genome Project
ENCODE: Encyclopedia of DNA Elements
EUR: European population from 1000 Genomes Project
INDEL: Insertion / Deletion
SNV: Single Nucleotide Variant
TF: Transcription Factor
TFBS: Transcription Factor Binding Site
TFBS-ID: Transcription Factor Binding Site affecting INDEL
TGP: 1000 Genomes Project
TSS: Transcription Start Site
